# Determination of Current Flow Induced by Transcutaneous Electrical Nerve Stimulation for the Treatment of Migraine: Potential for Optimization

**DOI:** 10.3389/fpain.2021.753454

**Published:** 2021-12-06

**Authors:** Chris Thomas, Dennis Q. Truong, Kiwon Lee, Choi Deblieck, Xiao Michelle Androulakis, Abhishek Datta

**Affiliations:** ^1^Research and Development, Soterix Medical, Woodbridge, NJ, United States; ^2^Ybrain Inc., Seongnam-si, South Korea; ^3^Academic Center for Electroconvulsive Therapy (ECT) and Neuromodulation, University Psychiatric Center, University of Leuven, Leuven, Belgium; ^4^Neurology, Columbia VA Health System, Columbia, SC, United States; ^5^School of Medicine, University of South Carolina, Columbia, SC, United States; ^6^City College of New York, New York, NY, United States

**Keywords:** transcutaneous electrical nerve stimulation (TENS), migraine, optimization, non-invasive electrical stimulation, modeling

## Abstract

**Introduction:** Transcutaneous electrical nerve stimulation (TENS) for migraine involves the application of pulsatile stimulation through electrodes placed on the forehead to target the underlying trigeminal nerves. It is a simple, safe modality and has secured clinical approval in several markets including the European Union and the United States. Despite nearing almost 7 years of use (postclinical approval), the exact mechanism of action is not fully known. Guided by the need to stimulate the trigeminal nerves bilaterally, electrode dimensions are simply required to extend enough to cover the underlying nerves. The goal of this study is to examine induced current flow [magnitude and spatial distribution of electric field (EF)] and another driver of stimulation [activating function (AF)] due to TENS therapy for migraine for the first time. We further consider the effect of changing the electrode dimension and shape and propose a design modification to deliver optimal flow.

**Methods:** We developed the first ultra-high-resolution finite element (FE) model of TENS for migraine incorporating the target supratrochlear (ST) and the supraorbital (SO) nerves. We first simulated the clinically approved V-shaped geometry. We then considered three additional designs: extended V-shaped, idealized pill-shaped, and finally an extended V-shaped but with greater contact spacing (extended V-shaped +CS).

**Results:** Our findings revealed that the clinically approved electrode design delivered substantially higher mean current flow to the ST nerve in comparison with the SO nerves (Medial: 53% and Lateral: 194%). Consideration of an extended design (~10 mm longer and ~ 4 mm shorter) and a pill-like design had negligible impact on the induced current flow pattern. The extended V-shaped +CS montage delivered relatively comparable current flow to each of the three target nerves. The EF induced in the ST nerve was 49 and 141% higher in the Medial and Lateral SO nerve, respectively. When considering maximum induced values, the delivery of comparable stimulation was further apparent. Given the existing electrode design's established efficacy, our results imply that preferential targeting of the ST nerve is related to the mechanism of action. Additionally, if comparable targeting of all three nerves continues to hold promise, the extended V-shaped +CS montage presents an optimized configuration to explore in clinical studies.

## Introduction

Migraine headache is characterized by an intense pulsing or throbbing pain in one area of the head accompanied by nausea and/or vomiting and increased sensitivity to light and sound. Migraine is one of the most common neurological disorders, affecting 18.5% of the general population (1) and causing marked disability in many patients (2). Migraine is thought to be a central neurovascular disorder. Further, migraine headaches are likely generated in the trigeminovascular system (3) that can be activated by cortical spreading depression, which is responsible for the migrainous aura.

In terms of treatment, the efficacy of preventive antimigraine drugs is limited (4) and the most effective among them can have unpleasant side effects. Transcutaneous supraorbital neurostimulation (TSNS) or more commonly transcutaneous electrical nerve stimulation (TENS) is an effective treatment for episodic migraine. The United States Food and Drug Administration (FDA) first approved a TENS device to treat migraine in 2014 *via* the De Novo pathway. The device (trade name: Cefaly) was approved for use before the onset of migraine, as a prophylactic treatment for migraine headaches in adult patients. In Europe, clinical approval or European Conformity (CE) was likely obtained prior to 2014 based on the conclusion of the PREMICE trial and a postmarketing survey (same data were used for FDA approval) (5). While TENS technology has been used for the treatment of muscle pain for decades (6), the novelty, in this case, is the stimulation applied to the central cranium regions. Similar to peripheral application, patients can self-administer TENS for migraine application and titrate dosage as required because there is no potential for overdose, as there is no established safe dosage and usage. Further, since effects are considered short-term (rapid in onset and also in offset), patients may use TENS in an on-demand fashion.

**Figure 1:**
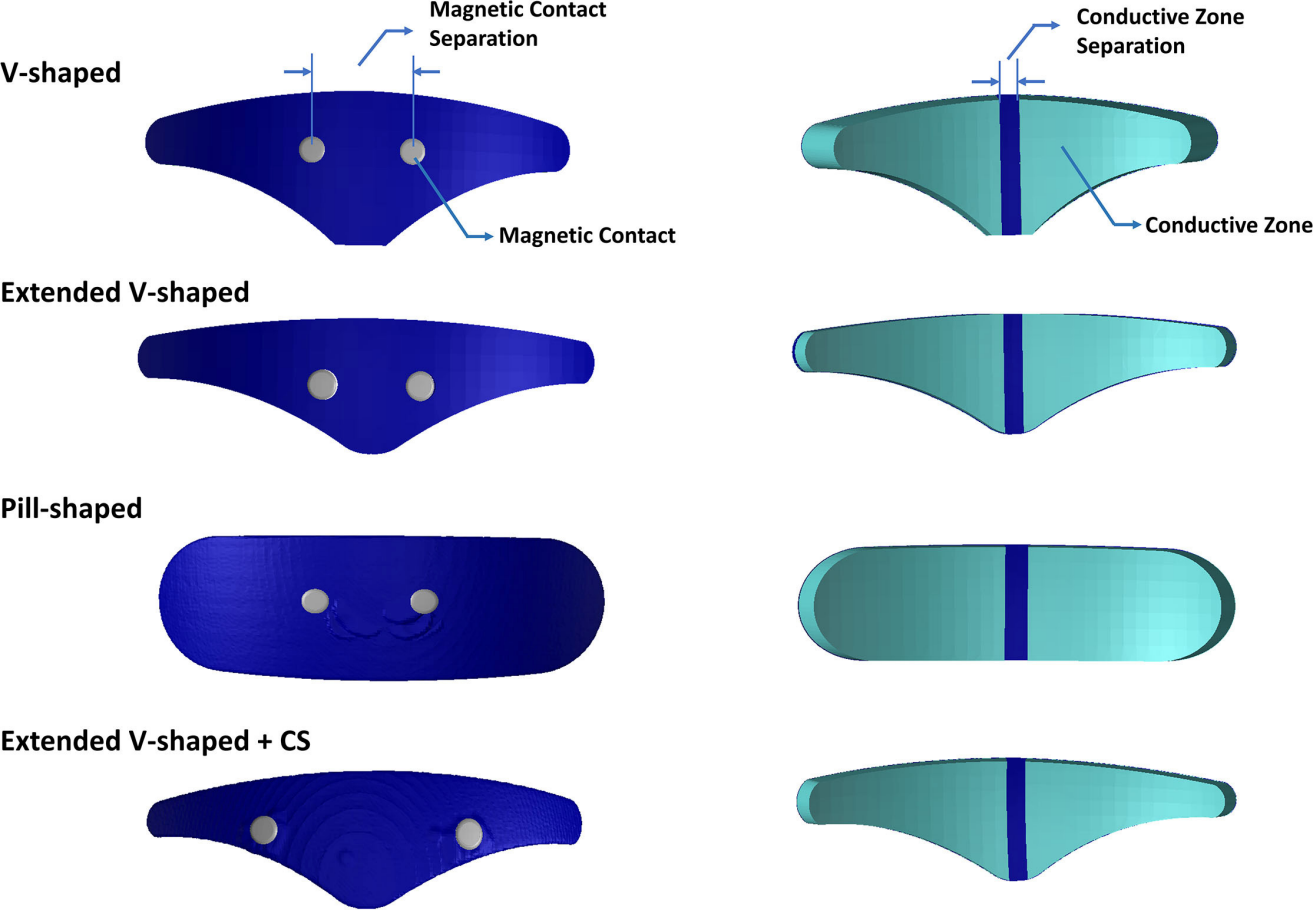
Electrode geometries evaluated in this study. Images on the left indicate the top surface. Images on the right indicate the bottom or patient contacting surface. The V-shaped geometry reflects the clinically approved design, and the extended V-shaped extends 10 mm wider but its surface contact area matched the clinically approved design. The pill-shaped geometry reflects an idealized design. The final geometry mimics the extended V-shaped design but has greater contact separation. Each geometry comprises two conductive zones on the bottom surface. The actual dimensions of all geometries are provided in Table 1.

The stimulation setup involves attaching a self-adhesive V-shaped electrode patch to the middle of the forehead by looking at a mirror. The device or the electrical pulse generator (EPG) is then attached to the two magnetic contacts (accessible only from the top surface of the electrode patch) *via* a magnetic connection (Figure 1). The EPG delivers current *via* this aforementioned patch to the intended target, that is, the trigeminal nerves. Since the first FDA approval in 2014, this technology has obtained approval for acute treatment and also for over-the-counter use. The stimulation duration is 20 or 60 min depending on preventative or acute treatment selection. The side effects are restricted to skin irritation, discomfort, sleepiness (<1%), dizziness, headache, and pain at the site of application and are fully reversible.

The mechanism of action is not fully known but is based on stimulating the upper branches of the trigeminal nerve to reduce the frequency of migraine attacks. Specifically, the conductive surface of the TENS electrode is intended to be wide enough to overlay the underlying supratrochlear (ST) and the supraorbital (SO) branches bilaterally (7).

Early studies indicated that stimulation in healthy volunteers results in a sedative effect (8). While how this translates as a therapeutic option for migraine pain remains unknown, it does validate that trigeminal nerve stimulation through the forehead can change CNS activity (5, 9). Moreover, this aforementioned sedative effect was found to have a particular dose-response, that is, a dose of 420 μC resulted in a sedative effect but not with 8.75 μC, despite the generation of strong paresthesia in both stimulation protocols. It is, therefore, assumed that TENS for migraine changes activity in the supraspinal centers associated with the pain matrix. This, as a consequence, increases the migraine threshold. Recent meta-analysis studies of randomized controlled trials indicate that TENS for migraine continues to be effective (10, 11). Specifically, reduction in migraine attacks, migraine days, headache days, and acute antimigraine drug intake were all found to be statistically lower, when using the device in prophylactic mode. Further, when used for acute remedy, visual analog scale-based measurement indicated a statistically significant difference at 1/2/24 h poststimulation (11). However, overall, these meta-analysis studies still advocate the need for additional studies with larger sample sizes to boost evidence and adoption.

It is to be noted that being an electrical stimulation technique, TENS-induced effects are naturally based on the applied electrical stimulus parameters, ranging from the waveform, frequency, intensity, duration, electrode configuration, etc. The application of current-controlled pulses for any TENS application (peripheral or central) indicates that due to physics, the total magnitude of current injected from one electrode is collected at the second electrode. Further, the usage of bipolar pulses for migraine application indicates no concept of electrode polarity as current reverses in the direction of every cycle. With respect to the exact current path or spread between the electrodes, this may not be necessarily trivial, dependent on the information that one is seeking to obtain. On a simplistic level, looking at the electrode separation, a shorter separation indicates restricted and shallow current flow. This is intentional as TENS is intended to activate underlying nerves as opposed to other electrical stimulation modalities (for instance, electroconvulsive therapy) that require greater separation as they require stimulating deeper brain structures. The aforementioned accepted concepts of electrical stimulation provide early insight into the current flow pattern due to the TENS for migraine electrodes.

Any electrical stimulation is dependent on electrode details (separation, shape, size, and materials) and underlying tissue information (anatomy and properties). The V-shaped electrode patch essentially reflects approximately two triangular-shaped electrodes (representing two conductive zones) spaced at ~5 mm apart (Figure 1). This implies the motivation for a largely restricted current flow pattern. Further, the circular magnetic contacts that serve as points of current ingress or egress are spaced at a relatively short distance dictated by the design of the EPG enclosure and its terminals. One would expect the minimal impact from the contact separation distance as the hydrogel interface would disperse the current delivery over the underlying ST and SO nerves bilaterally. However, to date, the electric field (EF) generated by TENS has not been systematically studied and therefore verified. This limits our ability to help to understand the mechanisms and potentially optimize stimulation.

The objective of this study was to determine the EF induced by TENS for migraine application and to further explore the role of electrode shape and contact placement. With respect to electrode shape, we consider the same V-shaped FDA-approved version but a wider design and a contrasting idealized “pill-shaped” version. The interest in the former was to facilitate increased current traversal through the more Lateral SO branches and in the latter to explore whether the choice of shape could play a role in the induced current flow pattern. For contact placement, we were interested in exploring whether increasing the separation would have any impact given the dispersion due to the hydrogel layer making final contact with the patient's skin.

## Methods

### Model Geometry

**Figure 2:**
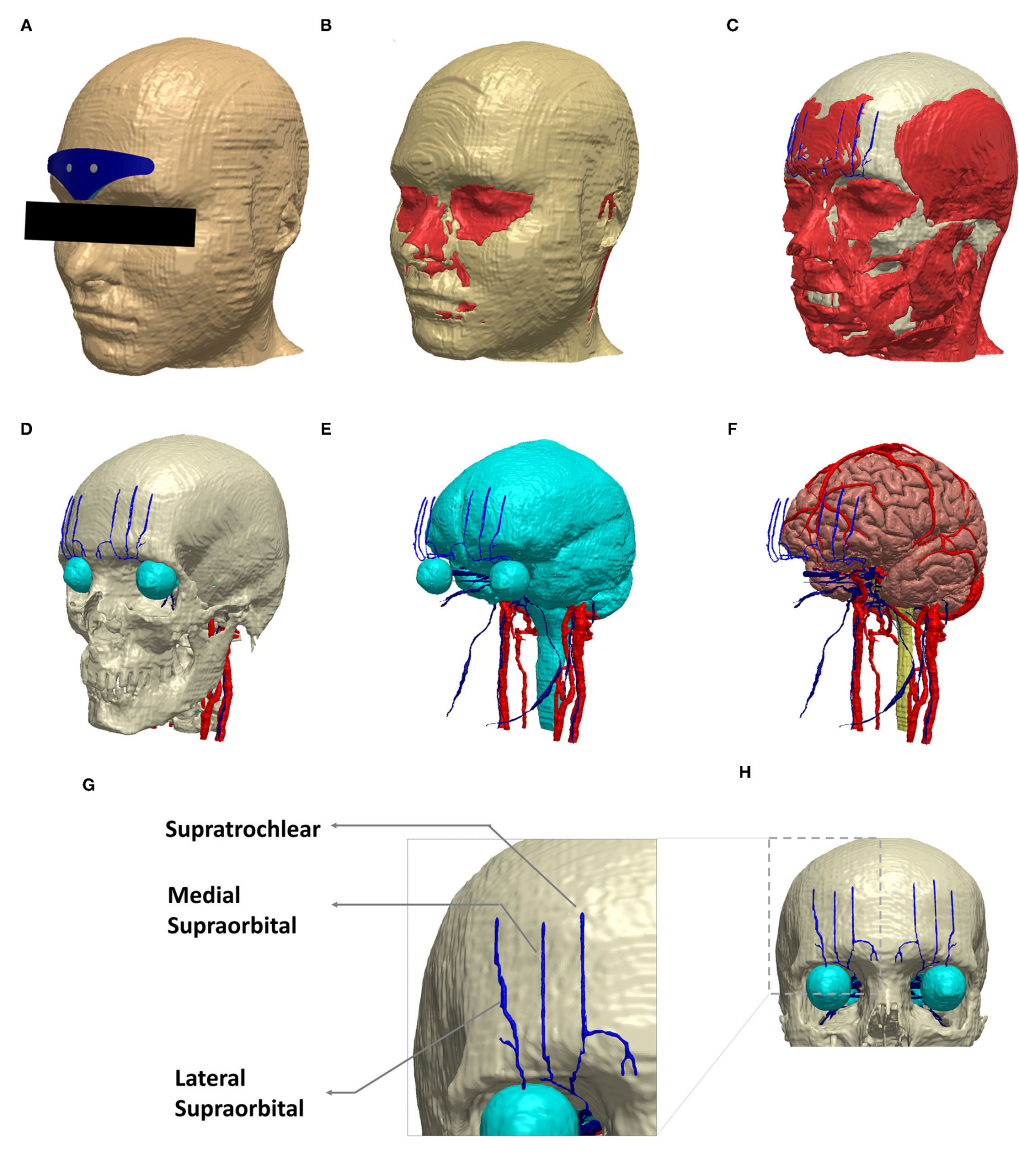
Schematic view of the implemented model geometry. Exemplary segmentation masks are shown: **(A)** scalp and the V-shaped geometry, **(B)** fat and muscle in red, **(C)** muscle along with skull (white), **(D)** skull and the target trigeminal nerves (blue). The eye region (part of CSF mask) and other cranial nerves (red) visible at the skull level are all shown. **(E)** CSF along with trigeminal and other cranial nerves. **(F)** gray matter, trigeminal nerves, and other cranial nerves. **(G)** The dashed section in **(H)** is expanded to highlight the trigeminal nerve geometry incorporated in the model. **(A–F)** were generated in the same perspective.

The head model used in this study was derived from an ultra-high-resolution (0.5 mm isotropic) head and neck dataset (MIDA: multimodal imaging-based detailed anatomical) available through the ITIS Foundation (12). We first processed the nifti (.nii) color masks from the MIDA model in MATLAB to recreate the segmentation masks. These masks were then imported into Simpleware (Synopsys Ltd., CA, United States), and any inaccuracies (continuity, overlap, and anatomical details) were corrected using inbuilt processing filters (13). Finally, tissue masks with similar electrical conductivity and not subject to any individual analysis were merged to simplify the meshing process and thereby reduce computation time (Figure 2).

Supratrochlear and supraorbital nerve compartments were manually created, guided by BioDigital Human, an interactive 3D software platform for visualizing human anatomy (14). In addition, a histomorphometric anatomical study was used for overall verification of the anatomical location of the nerve compartments (15).

### Electrode Configuration

The electrode configurations (or patches) were modeled as computer-aided design (CAD) models (STL files) in SolidWorks and imported into the image dataset space. This enabled interactive positioning, that facilitates the mimicking of the actual resting position when used clinically (i.e., above the eyebrow and lower edge aligned with the horizontal line joining eyebrows). The electrode patches reflected realistic composition comprising of a multipart arrangement of the plastic backing, carbon layer, and a patient contacting hydrogel layer. The contacts were immersed in the electrode geometry and, similar to the actual physical version, were accessible only from the top layer (Figure 1). Each patch consisted of two electrodes or two conductive zones separated by an insulating material. The exact dimensions are noted in Table 1.

**Table 1:** Electrode dimensions and contact separation.

	**Width (mm)**	**Height (mm)**	**Depth (mm)**	**Conductive zone separation (mm)**	**Contact separation (mm)**
V-shaped	94	30	2.2	5	13
Extended V-shaped	104	26.7	2.2	5	13
Pill-shaped	104	26.7	2.2	5	13
Extended V-shaped +CS	104	26.7	2.2	5	43

*The V-shaped geometry reflects the clinically approved design. The conductive zone separation was the same across all montages. The contact separation was the same for the first three electrode geometries. The dimensions do not capture the actual shape. Refer to Figure 1 as well*.

The following four electrode configurations were simulated:

1. **V-shaped**: This configuration reflected the classic clinically approved design.

2. **Extended V-shaped**: Same shape as the clinically approved version but with width extended by 10 mm. The height was accordingly reduced to ensure the same contact area.

3. **Pill-shaped**: This design reflected an idealized shape using the same width and height of the extended V-shaped design.

4. **Extended V-shaped** with greater contact separation (**extended V-shaped +CS**): The aforementioned configurations shared the same contact separation. In this version, contact separation was set to 43 mm.

### Meshing

The combined geometries (tissues and electrodes) were converted to masks, and any overlap or unintended empty voxels were accordingly revised using a combination of filters and Boolean operators. We then used the multipart adaptive meshing feature and set a coarseness parameter of −10 for the nerves and −16 for all other compartments. The setting of the coarseness parameter automatically controls the minimum and maximum edge length and target maximum error. The exact settings are listed in Table 2. This was motivated by the need to preserve small features in the thin nerve regions by generating a denser mesh as opposed to the other regions. The final generated meshes were exported into COMSOL Multiphysics (Burlington, MA, United States) for performing FE analysis.

**Table 2:** Mesh properties of the FE model.

**Setting**	**Coarseness**	**Target minimum edge length (mm)**	**Target maximum error (mm)**	**Maximum edge length (mm)**	**Surface change rate**
Trigeminal nerves	−10	0.6	0.05	1.4	20
All other tissue compartments	−16	0.81	0.05	1.94	32

*The target trigeminal nerve regions were set to a different meshing parameter than all other compartments. This ensured a denser mesh creation such that small features in the regions remain preserved to maintain accuracy*.

### FE Model

A transcutaneous electrical nerve stimulation device approved for migraine treatment generates a biphasic, rectangular, symmetrical waveform with a phase duration of 250 μs and an interphase duration of 5 μs. The frequency of stimulation is 60 Hz. Upon the stimulation initiation, the current intensity delivered increases gradually for the first 14 min until it reaches a maximum of 16 mA. It is to be noted that tissue properties consist of both real (conductive) and reactive (permittivity) components. However, for lower frequencies (~<10 kHz), the real component dominates (16). Since the waveform frequency in TENS is low (60 Hz), it can be assumed that our medium (geometry) consists only of conducting material. Therefore, with respect to electrical conductivities, representative isotropic average values were assigned to different tissue compartments and electrode materials (Table 3).

**Table 3:** Assigned electrical conductivities.

**Tissue compartment / electrode material**	**Electrical conductivity (s/m)**
Scalp	0.465
Fat	0.04
Muscle	0.35
Blood	0.7
Skull	0.01
CSF	1.65
Gray matter	0.276
White matter	0.126
Nerves	0.126
Air	1 e-7
Hydrogel	0.1
Magnetic contact	9.8 e5
Plastic backing	1.667 e-16
Carbon layer	10

*Representative isotropic electrical conductivities assigned to the different tissue compartments and the electrode material. The conductivity value of hydrogel reflected the same hydrogel material that is used with the clinically approved electrode. Typical values are chosen for the other compartments*.

Further, under quasi-static approximation, the EF in the volume conductor is determined by deploying the classic Laplace equation as time variation in the electric signal is not considered (17, 18). Additionally, since the device's EPG serves to deliver current through the two contacts, the boundary condition is essentially set at the contacts. As a result, the EPG itself is not considered in the final simulation geometry.

The following boundary conditions are imposed: (1) an inward normal current density corresponding to the highest intensity generated by the device (16 mA) at one contact, (2) ground applied at the second contact, and (3) all other external surfaces treated as insulated. The conjugate gradient solver was used. The final FE models on average comprised >30 million elements with >40 million degrees of freedom.

### Data Analysis

**Figure 3:**
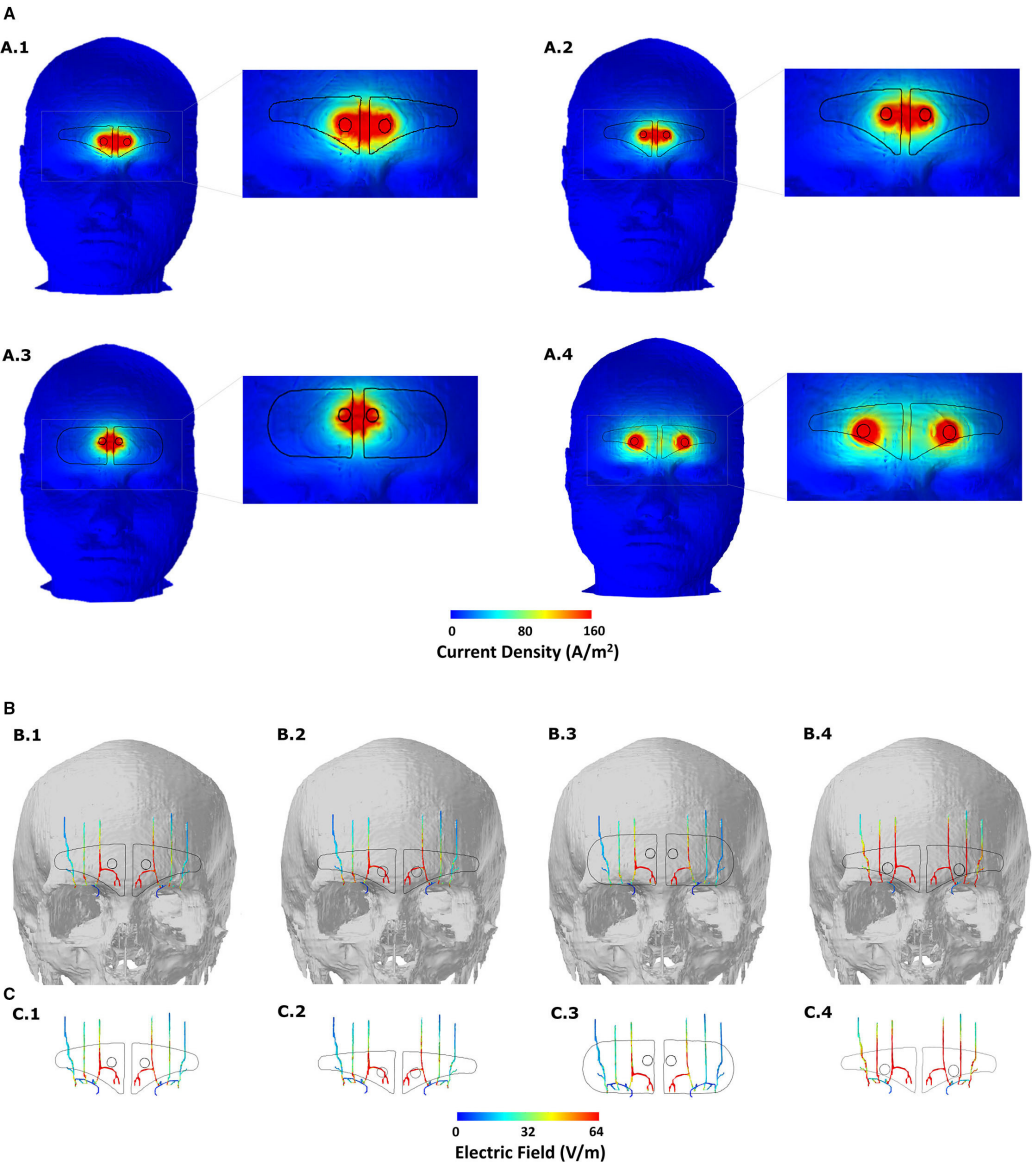
Induced current flow pattern for each of the four-electrode configurations **(A)** Scalp current density plots. The dashed section in each image is expanded in the immediate right to better elucidate current flow patterns. Current shunting dominates across the two contacts for the first three montages and contact separation distance plays a defining role in the overall current flow pattern. Increasing the contact separation results in individual current concentration at the location of the two contacts reflecting less shunting and more inward flow into the underlying tissues. **(B)** Induced EF on the surface of the target nerves. The skull mask is included in the background to help related flow to the anatomical location. **(C)** Same and **(B)** but without any background. All EF images are plotted to the mean induced value on the ST nerve due to the V-shaped or clinically approved design (see Table 4).

**Figure 4:**
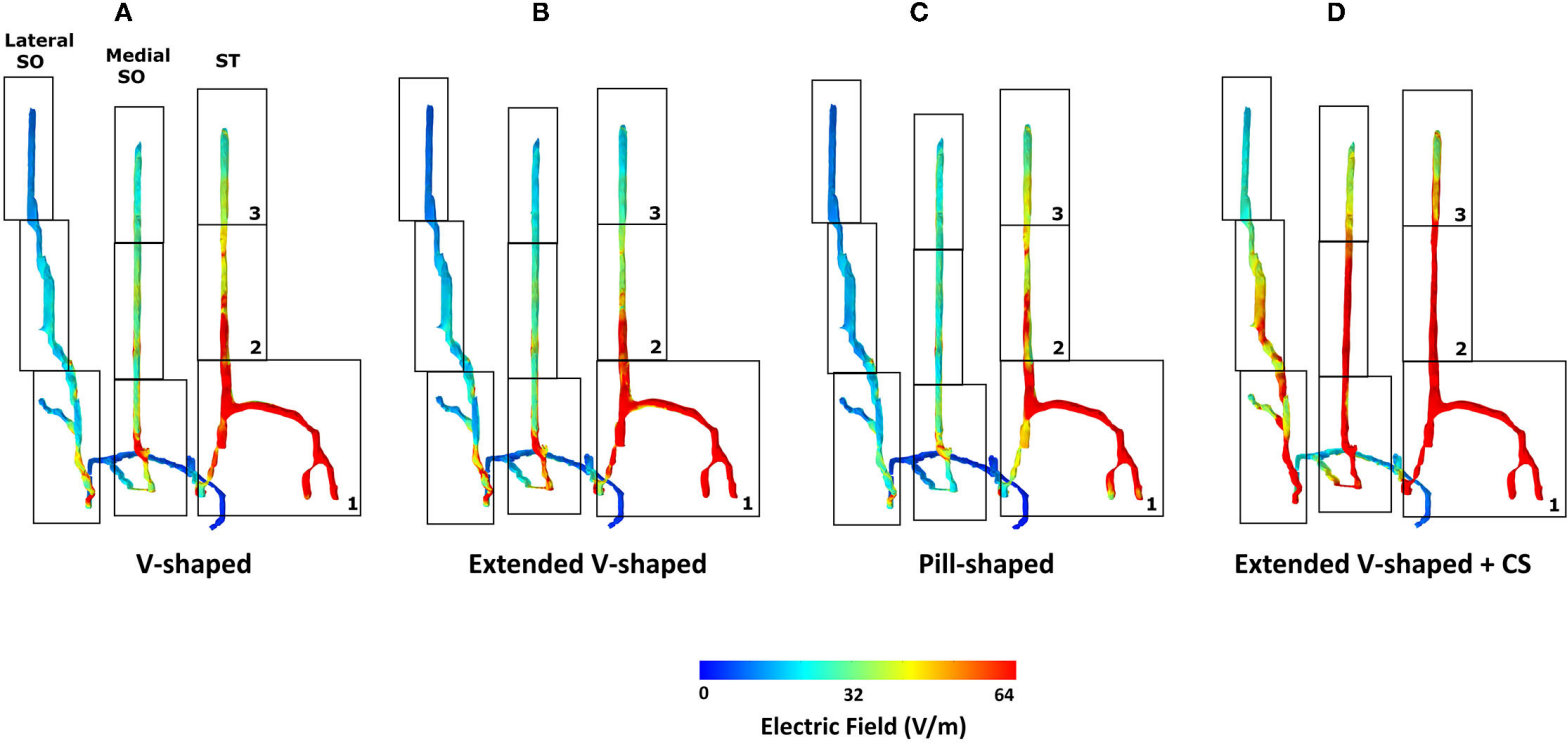
Detailed analysis of induced flow on the left trigeminal nerves. We partitioned each of the individual target nerves (ST, Medial SO, Lateral SO) into three subsections (1, 2, and 3) to facilitate easier comparison across the four montages. The extended V-shaped +CS configuration results in more comparable current flow in each of the three target nerves. For the first three montages, we did not notice any marked difference in the overall flow. Given symmetric patch placement, we expect the same observation in the right trigeminal nerves.

**Figure 5:**
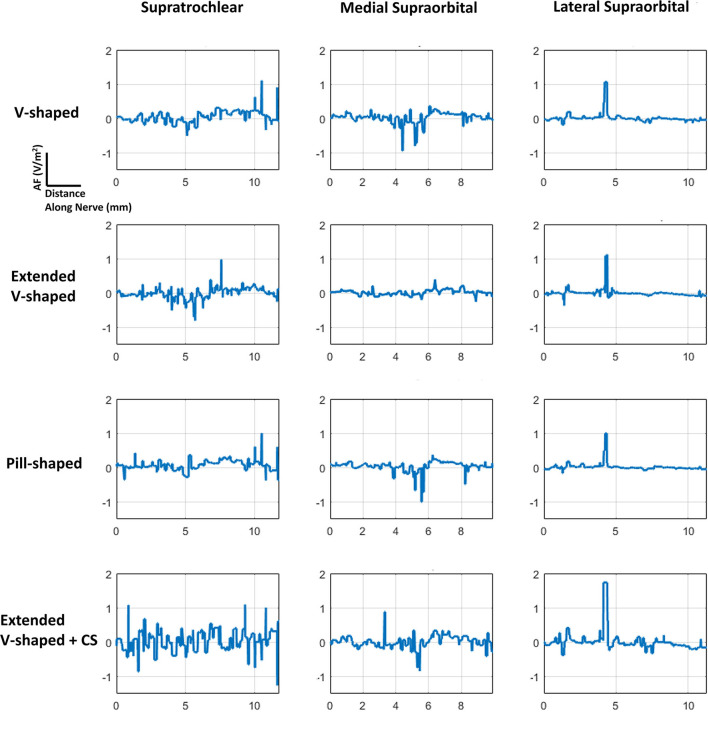
AF plots for each of the configurations simulated. Since each nerve had a dominant orientation (inferior to superior), plots were generated along a single dimension. Further, a straight section of the nerve located approximately at the middle of the electrode was considered for each of the individual nerves. The same section was used when comparing across the montages. The plots were also normalized with respect to the induced values due to the V-shaped design to facilitate easier comparison.

We first generated skin surface current density magnitude plots to help to elucidate the extent of spatial spread and related current shunt (or current loss) before current enters the underlying tissues (Figure 3). Subsequently, we generated surface EF plots for the target nerves. These plots help to determine the magnitude, extent, and importantly whether each one of them receives equivalent current flow (Figures 3, 4). To quantify the extent of uniform current delivery and potentially related nerve recruitment, we determined the percentage of current flow delivered to the ST nerve in comparison with the SO nerves (Table 4). Finally, we considered the nerve activating function (AF) to help to highlight differences among configurations with respect to a neural excitability metric (Figure 5).

**Table 4:** Induced EF, current density, and percentage increase of EF induced at ST nerve with respect to the other nerves.

		**Electric field (V/m)**	**Current density (mA/cm^**2**^)**	**Electric field (V/m)**	**Current density (mA/cm^**2**^)**	**Electric field (V/m)**	**Current density (mA/cm^**2**^)**	**(ST-medial)/medial**	**(ST-lateral)/lateral**
		**ST nerve**		**Medial SO**		**Lateral SO**			
V-shaped	max	274.311	3.456	147.981	1.865	84.532	1.065	85.37%	224.50%
	mean	63.890	0.805	41.555	0.524	21.698	0.273	53.75%	194.46%
	median	51.458	0.648	35.716	0.450	18.547	0.234	44.07%	177.45%
Extended V-shaped	max	355.326	4.477	260.080	3.277	144.308	1.818	36.62%	146.23%
	mean	70.061	0.883	44.465	0.560	22.320	0.281	57.56%	213.89%
	median	58.015	0.731	35.743	0.450	16.974	0.214	62.31%	241.78%
Pill-shaped	max	248.810	3.135	105.900	1.334	66.362	0.836	134.95%	274.93%
	mean	58.395	0.736	33.029	0.416	17.564	0.221	76.80%	232.47%
	median	47.250	0.595	30.174	0.380	15.317	0.193	56.59%	208.48%
Extended V-shaped +CS	max	411.711	5.188	311.699	3.927	206.617	2.603	32.09%	99.26%
	mean	115.348	1.453	77.101	0.971	47.836	0.603	49.61%	141.13%
	median	107.720	1.357	70.185	0.884	39.922	0.503	53.48%	169.83%

*Across all montages, the ST nerve resulted in the highest induced values, followed by the Medial SO and the Lateral SO nerve. The extended V-shaped +CS not only delivers the highest current flow to target regions but also ensures comparable flow. Max refers to the maximum*.

## Results

For each of the four configurations, we investigated the induced current flow and the AF for the individual trigeminal nerves. Figure 1 illustrates the electrode geometries considered for the study. The V-shaped geometry mimics the exact physical dimensions of the clinically approved version. All versions employed similar overall electrode composition (plastic backing, magnetic contacts, and hydrogel layer). Figure 2 depicts the implemented 3D model geometry and the tissue masks are considered. Further, the 3D reconstruction of the trigeminal nerves reflects accurate replication of actual anatomy (location, orientation, length, and thickness).

The clinically approved electrode montage (V-shaped) revealed greater current shunting across the scalp with respect to the current flowing into the underlying structures (Figure 3A, A.1). This is verified by the corresponding nerve EF plot (Figure 3B, B.1) that reveals that the assumed current dispersion due to the hydrogel layer is not sufficient enough to optimally deliver current flow to all the nerves bilaterally. While the ST nerve is subjected to the highest EF as a direct consequence of the restricted current flow at the level of the skin, the Medial and the Lateral SO nerves receive substantially lesser current flow. As mentioned above, dominant current shunting is expected by design, given the electrode montage choice. This is guided by the anatomical location of the target trigeminal nerves that exist superficially within the fat layer (19), so the intent is to naturally generate a shallow current flow pattern.

The consideration of an extended V-shaped design does not substantially alter the current flow pattern at the level of the skin or trigeminal nerves (Figures 3A, A.2, 3B, B.2). So, an increase of ~12 mm width (but with height reduced to match contact area to the clinically approved version) does not result in any noticeable advantage in targeting the trigeminal nerves. The simulations using the pill-shaped design also indicate the same dominant current shunting at the skin and inadequate dispersion to uniformly target the three trigeminal nerves (Figures 3A, A.3, 3B, B.3). Our simulations, therefore, highlight a key finding that is not necessarily understood unless one incorporates computational modeling in the device development process, that is, the contact separation dominates current flow over the extent of the current dispersion due to the patient contacting hydrogel layer.

The simulations due to the extended V-shaped + CS montage verify the critical role of contact separation. At the level of the skin, we observe two independent concentrations, reflecting preferential current entry or exit at the location of the two contacts (Figure 3A, A.4). This results not only in an overall more dispersed current flow (i.e., not restricted to between the contacts) but also increased current flow into the underlying structures. Therefore, as a consequence, simulations indicate increased current flow into the wider Medial and the Lateral SO nerves, such that all three nerves are targeted similarly (Figures 3B, B.4, 3C, C.4).

The inspection of current flow by compartmentalizing each of the nerves into three sections helps to further evaluate any subtle difference across the montages (Figure 4). Across the montages, it is clear that the ST nerve is subjected to the highest EF, followed by the Medial and then the Lateral SO nerves, largely dictated by the proximity to the contacts. Further, the inferior (lowermost) or compartment 1 is subjected to the highest EF, followed by the middle and then the superior compartment, across all montages. Due to the slightly shorter height of the extended V-shaped montage (~4 mm) with respect to the V-shaped montage, we note a marginally lower EF in the superior compartment of the ST nerve (Figure 4B). However, the additional width (~10 mm) of the extended V-shaped design does not translate into any noticeable difference in the current flow pattern in the outer Medial and Lateral SO nerves. Further, we did not notice any marked difference between the pill-shaped and the two V-shaped montages. For the extended V-shaped +CS montage, we observed prominent current flow along the entire length of the ST nerve, in the inferior (compartment 1) and middle (compartment 2) and a portion of the superior (compartment 3) of the Medial SO nerve, and in the inferior and middle compartments of the Lateral SO nerve. We quantify the actual induced values in a tabular format (Table 4) and note values at the ST nerve with respect to the SO nerves. Considering mean values, the EF induced in the ST nerve can be 53–76% higher than the Medial SO nerve and 194–232% higher than the Lateral SO nerve across the first three montages. The consideration of the extended V-shaped +CS montage indicates 49 and 141% higher values with respect to the Medial SO and Lateral SO nerves, respectively. The delivery of comparable stimulation is made more apparent when considering the maximum induced values. Additionally, the extended V-shaped +CS montage not only delivers the highest current flow to target regions for the same injected current (16 mA) but also ensures a more comparable flow.

The consideration of the AF plots for each of the montages largely reflects similar profiles for any particular nerve (Figure 5). This is expected as the AF provides a relative estimate of nerve excitation based on the extracellular EF. Further, owing to the same bipolar electrode montage in each case with differences with respect to shape and contact spacing (for one montage), the overall current flow pattern is similar. This results in a similar voltage profile, resulting in a similar EF pattern and ultimately resulting in a similar AF profile. The clear difference is the expected highest magnitude of AF for the extended V-shaped +CS montage across all nerves.

## Discussion

The purpose of this study was to develop an understanding of the current flow patterns induced due to the FDA and CE-approved TENS intervention for migraine treatment. While the default V-shaped design is intended to target the underlying trigeminal nerves, to date, there has been no attempt to verify whether this is the case. We leverage the use of FE-based simulation using an ultra-high-resolution multimodal dataset to systematically analyze current flow patterns and related nerve excitation. The need for a submillimeter resolution dataset was motivated specifically because of the requirement of incorporating tiny target structures of interest (trigeminal nerves). We further considered the impact of different electrode shapes and propose a design modification to deliver optimized current flow. The geometry used for the final analysis reflected realistic head anatomy including nerves and mimicked actual electrode design, components, and dimensions.

Our simulations reveal that contrary to assumption, the default V-shaped electrode does not deliver comparable (or equivalent) current to the underlying target nerves. Specifically, the ST nerves are subjected to more current than the Medial and Lateral SO nerves. Given, TENS for migraine is widely accepted as an efficacious technique, and this may indicate that stimulating the ST nerves preferentially is sufficient for its mode of action. It could also indicate that despite its efficacy record, stimulation could be further optimized by enhancing the recruitment of the SO nerves. Given limited neurophysiological studies (5) and related lack of understanding of the mechanism of action, it is rational to consider maximizing currents in target regions (13), as regions with higher EF are more likely candidates for the stimulation.

We note that for the electrode approach employed for migraine treatment (materials and design), contact separation predominantly determines induced current flow pattern as opposed to shape (extended V-shaped or idealized). Further, this principal role of the contact separation is likely made prominent due to the limited extent of current dispersion observed due to the hydrogel layer. While not the focus of this study and non-trivial to speculate unless simulated, a range of factors such as the distance between the conductive zones (~5 mm), hydrogel conductivity, and thickness of the hydrogel layer is likely to play a role.

Our simulations also provide supplementary safety information. Clinical study and regulatory concerns about any non-invasive electrical stimulation generally focus on current density and related charge and power density (calculated by factoring in time and voltage, respectively) (26). As the current density estimate at the target is not readily available, it is convenient to report electrode surface current density. Given the suprathreshold pulse delivery of TENS, the safety limits proposed by the studies of Agnew and McCreery (20) and McCreery et al.(25) are most applicable. Specifically, a current density limit of 25 mA/cm^2^ is considered safe as values below the limit were shown to not induce tissue (brain) damage. Our simulations indicate a maximum current density of 3.45 mA/cm^2^ at the ST nerve using the clinically approved V-shaped montage and 5.18 mA/cm^2^ using the optimized montage, values that are well under the safety limit. These values substantiate the safety of the technique.

It should be noted that while our simulations assumed a maximum allowed current intensity (16 mA), induced EF at a lower injected scalp intensity can be calculated assuming a linear relationship. This follows the quasi-static field approximation in our modeling process that implies linearity of the induced solution (17, 18, 21).

The prediction accuracy of any FE modeling such as this is contingent on the precise representation of anatomy along with electrode detail and compartment properties. While our model captures essential anatomical details with the simulation methodology already validated (22, 23), additional technical improvements could be pursued, contingent on the question to be answered. Our nerve tissue reflects a single compartment with a single electrical conductivity and is devoid of any fascicle information. Enhanced metrics of neural excitation may be predicted by directly coupling induced EF data to biophysical models of nerves (24). However, given our goal to determine overall current flow and study impact due to different montages, considered model details are adequate.

In closing, a clinical validation study employing the extended V-shaped +CS design would be needed to be planned to determine whether it manifests into better outcomes. Irrespectively, the development of the first-ever model TENS model for migraine will help support advances, from the exploration of additional electrode designs, considering individual anatomy, and study the extent of variation in current flow, to incorporate additional model detail to continue to improve prediction accuracy.

## Data Availability Statement

The original contributions presented in the study are included in the article, further inquiries can be directed to the corresponding author.

## Author Contributions

CT and AD developed the concept idea. CT, DT, KL, CD, XA, and AD performed the literature review, planned the candidate electrode montages, and edited the main manuscript. CT and AD performed the e-field modeling for the four montages. All authors contributed to the article and approved the submitted version.

## Funding

The work performed in the study was partially funded by grants to AD from NIH (75N95020C00024) and DoD (W81XWH-20-P-0016).

## Conflict of Interest

CT, DT, and AD are employees of Soterix Medical. KL is an employee of Ybrain. The remaining authors declare that the research was conducted in the absence of any commercial or financial relationships that could be construed as a potential conflict of interest.

## Publisher's Note

All claims expressed in this article are solely those of the authors and do not necessarily represent those of their affiliated organizations, or those of the publisher, the editors and the reviewers. Any product that may be evaluated in this article, or claim that may be made by its manufacturer, is not guaranteed or endorsed by the publisher.
